# Developmental Physiology: Grand Challenges

**DOI:** 10.3389/fphys.2021.706061

**Published:** 2021-06-10

**Authors:** Warren Burggren

**Affiliations:** Developmental Integrative Biology Research Group, Department of Biological Sciences, University of North Texas, Denton, TX, United States

**Keywords:** DOHaD, developmental plasticity, climate change, toxicology, epigenetics, heterokairy, allometry, animal models

## Introduction

The field of Developmental Physiology faces both great opportunities and significant challenges in the years ahead. While this provides “job security” for new investigators in this area, it also requires delineating some of the major challenges to maximize progress. I suggest that pressing challenges in Developmental Physiology fall into three categories: demonstrating broad relevance, promoting conceptual advances and improving experimental approaches.

## Challenge: Demonstrating Broad Relevance

### Developmental Origins of Health and Disease

**Figure 1:**
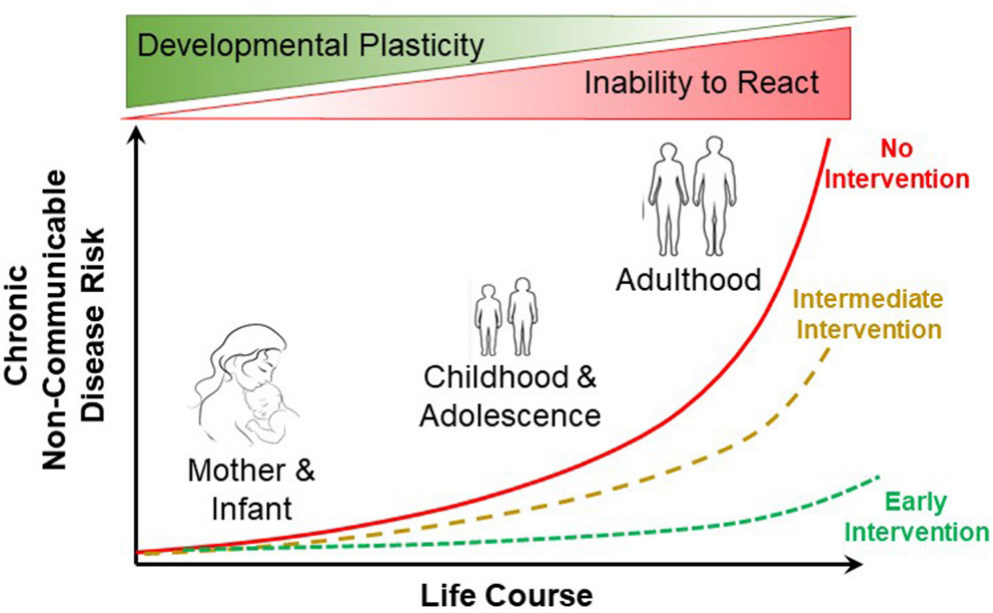
Influence of early life course intervention on the risk of non-communicable disease in humans. Early intervention (e.g., improved maternal and neonatal nutrition) can result in greatly reduced risk of non-communicable disease in adulthood. Modified from Baird et al. (2017).

The developmental origins of health and disease (DOHaD) encapsulates how environmental stressors in early development can lead to subsequent health implications in adult humans (Figure 1). DOHaD is now influencing many fields of research (Suzuki, 2018), including epidemiology (Heindel et al., 2017), epigenetics (Safi-Stibler and Gabory, 2020), specific pathophysiologies (Arima and Fukuoka, 2020; Briana and Malamitsi-Puchner, 2020) and policy and public health (Loi et al., 2013), to name but a few such fields. Among the many challenges for human-oriented studies in DOHaD is translating what we learn from developmental studies into actionable clinical and public health practices (Hanson et al., 2019). What is increasingly understood is how early developmental trajectories can be programmed by early developmental events. Yet, adult disease prevention through intervention in early development in many instances lags behind the more common (and far more challenging, not to mention expensive) practice of treating diseases in adults arising from, as but one example, maternal or neonatal nutrition (Baird et al., 2017). In basic animal physiology, the consequences of early life conditions for adult experimental animals are not generally appreciated (or are ignored), judging from the consistent lack of any mention of rearing conditions in published physiology articles using adult animal models. Recognizing how early life conditions alter adult physiology remains a challenge for the field.

### Developmental Physiology and Climate Change

Great effort is being expended to determine the effects of projected climate change on individual organisms all the way up to the ecosystems they inhabit. All too often, however, studies singularly involve adults of a species. Far less studied are effects in developing animals of temperature change, ocean acidification and other climate change-related phenomena. This exposes a truism: there will be no adults of a species to experience climate change effects if the species' offspring do not develop correctly (or at all) in the first place! Indeed, climate change presents numerous possible stressors that could affect development in a similar manner to DOHaD-related stressors during the developmental process (Kingsolver and Buckley, 2020; Sanger, 2021). One of the challenges for Developmental Physiology, then, is to emphasize and analyze effects of climate change on early life stages and how this ultimately influences species fitness.

### A Deeper Understanding of Toxicological Effects During Development

The early life stage approach advocated above has been exploited in toxicology and the study of environmental contamination. A prime example of this is the April 2010 DeepWater Horizon oil spill in the Gulf of Mexico, where considerable focus has been on the effects of oil exposure in developing fish and birds. What emerges is that early life stages have often higher vulnerability to environmental toxicants than at later stages (Incardona and Scholz, 2018; Pasparakis et al., 2019). Yet, what is also becoming clear is that organisms can develop considerable resilience to toxicants that can transfer to their offspring through non-genetic mechanisms (Vandegehuchte and Janssen, 2014; Kishimoto et al., 2017; Bautista and Burggren, 2019). A challenge, then is to understand the limits and mechanisms underlying inheritance of this toxicant resistance and how this might alter both science and policy involving environmental contamination.

## Challenge: Promoting Conceptual Advances

### Expanding the Role of Epigenetics

Mendelian genetics is increasingly inadequate in explaining key developmental phenomena ranging from misdirected gene expression resulting in adult disease states to non-genetically inheritance of modified phenotypes. We now know that, typically, the modification of DNA expression in germ or somatic cells alike that produces modified phenotypes involves “readers” that assess the state of epigenetic markers on genes. Subsequently, “writers” and “erasers” modify the state of epigenetic markers according to the prevailing environment and thus modify expression of genes that these markers regulate. The result is altered phenotypes within or across generations (Walker and Burggren, 2020; Wan et al., 2021; Wei and He, 2021). Essentially, developmental phenotypic plasticity, one of the basic principles of developmental physiology (Burggren, 2020a) and discussed above in the context of DOHaD, derives largely from the writing and erasing of epigenetic markers. A challenge for developmental physiologists is to integrate, from molecular to organismal level, the mechanisms and outcomes associated with epigenetic reading, writing and erasing and the associated changes in gene expression, both within and across generations.

### Physiological Heterochrony and Heterokairy

**Figure 2:**
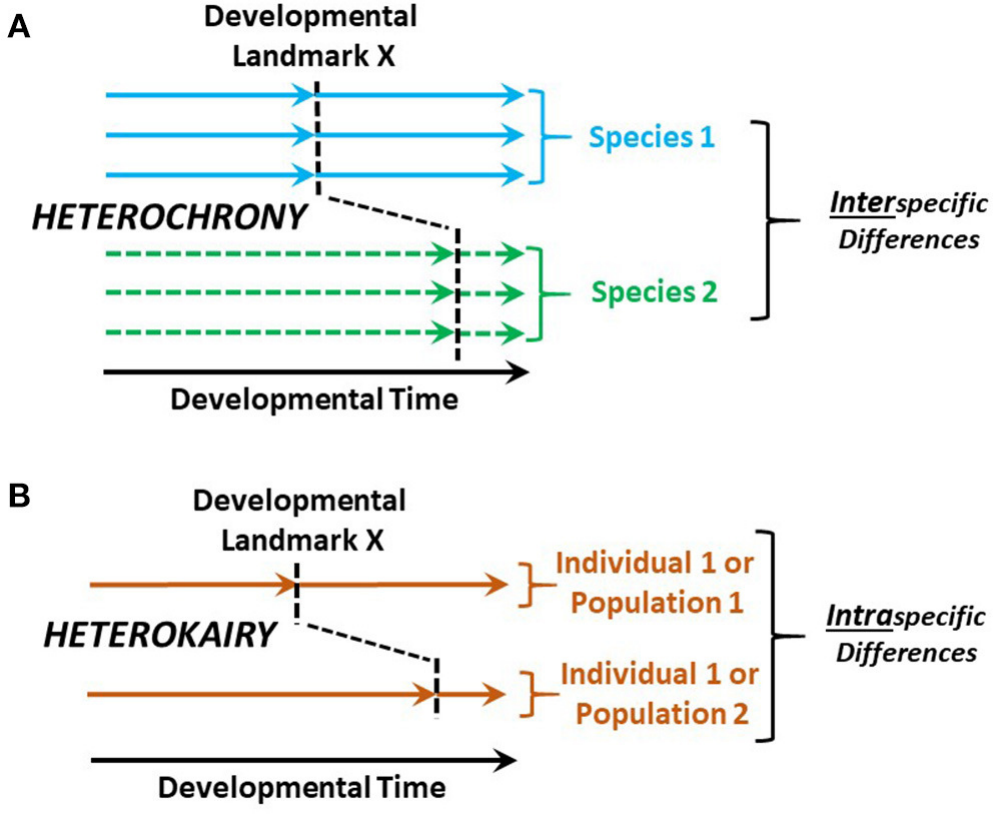
Heterochrony compared with heterokairy. **(A)** Heterochrony compares the timing of developmental landmark between a species of interest and the ancestral species. **(B)** In contrast, heterokairy compares the developmental landmarks of two different individuals or populations within a single species. Typically, the developmental landmarks are anatomical—e.g., the appearance of gill arches, cardiac valves or nephrons. However, these concepts apply equally well to physiological developmental landmarks such as the onset of gill ventilation, the onset of anterograde blood flow through the heart, or the first production of urine.

Heterochrony is the change in timing of developmental landmarks in a species compared to its ancestral species (Keyte and Smith, 2014). As a companion concept, heterokairy focuses on the intraspecific change in timing of developmental landmarks in an individual or a population (Figure 2). Like heterochrony, the concept of heterokairy is proving similarly useful in understanding developmental processes (Spicer and Burggren, 2003; Rundle and Spicer, 2016; Spicer et al., 2018). However, both of these concepts are typically employed to consider anatomical development with typically less focus on physiological processes. Yet, both concepts also equally apply to physiological development. The challenge for developmental physiologists, then, is to more deeply investigate heterochrony and especially heterokairy, allowing important insights into phenomena such as developmental phenotypic plasticity and the contributions of physiological processes to fitness during development.

### Allometry and Development

The allometry of development has long been of interest to developmental physiologists (Gould, 1975; Weder and Schork, 1994; Stern and Emlen, 1999; Singer and Mühlfeld, 2007; Vea and Shingleton, 2021). However, a fundamental challenge that remains unresolved involves reconciling two basic yet conflicting tenets of allometry and development (Burggren, 2020b). Thus, a basic tenet of allometry is that animals of different sizes being compared should be in the same physiological state. Yet, a basic tenet of development is that developing animals are constantly changing physiological state. How then, can allometric analyses simplistically involve both a larva or fetus as well an adult? This is not an insurmountable problem, as there are likely to emerge (or, at least *should* emerge) weighted statistical approaches that take changing physiology into account as allometric analyses are performed (or vice versa).

## Challenge: Improving Experimental Approaches

### Seeing Development as a Continuum Rather Than Discrete Events

Traditionally, developmental physiology has been built upon individual studies carried out at a single or just a few discrete points in development. Yet, I posit that the most robust understanding of “how physiology develops” lies in considering a *continuum* from germ cells to organismal senescence (as encapsulated in the description of the Developmental Physiology Section of Frontiers in Physiology). Any attempted synthesis of developmental physiology gleaned from analysis of multiple physiological studies each conducted at only a single point of development will, at best, be tedious and, at worst, lead to a biased or inaccurate conclusion. By considering a continuum, physiological measurements can more readily be put in context of an organism's entire life cycle. Thus, a specific challenge is to promote experimental protocols that gather data along multiple points of the developmental continuum.

### Expanding (and Verifying) our Animal Models

The use of animal models is, of course, a stalwart of developmental physiological investigation. Whether the larvae of zebrafish or *Xenopus*, the chicken embryo, or the mouse or sheep fetus, animal models have been of major benefit to both enhance our basic understanding of the “biology of development” as well as provide information of translational importance to biomedical research. Additionally, invertebrate models including the fruit fly *Drosophila melanogaster* and the nematode *Caenorhabditis elegans* have, as animal models, provided great insight into development. Yet, the challenge is to not become complacent in our use of these animal models. As the Danish physiologist and Nobel Prize winner August Krogh declared, for every physiological question there is the ideal animal to answer it (Krogh, 1929). Initially successful animal models become entrenched in the research community, sometimes resulting in the lack of penetration or marginalization of alternative animal models that could prove to be superior in some respects (Flores Santin and Burggren, submitted).

### Experiments Incorporating Multiple Stressor Experiments and Stochasticity

**Figure 3:**
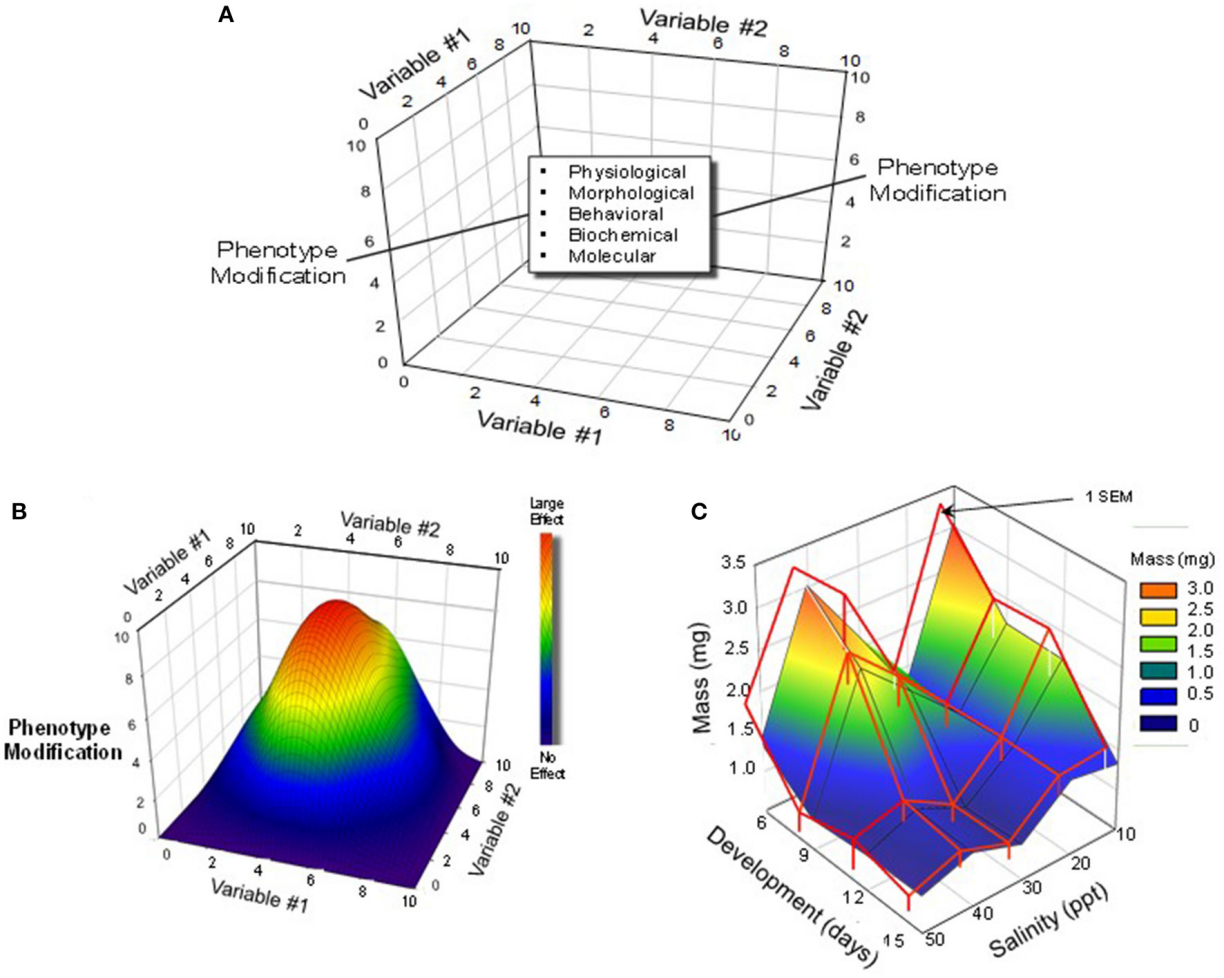
Multi-variable experimental designs and possible outcomes. **(A)** A three-dimensional schematic depiction of a protocol that would employ multiple levels/concentrations/doses of multiple variables. **(B)** A hypothetical outcome on a developing animals phenotype produced by simultaneous variation in two different variables. In this schematic, the largest effect is created by a combination of intermediate levels Variable #1 and Variable #2. Modified from Burggren and Mueller (2015). **(C)** An actual example of interactions between development (Variable #1) and rearing salinity (Variable #2) in the brine shrimp *Artemia franciscana*. Note that there was an unexpected bimodal effect of rearing salinity, with the largest effect appearing early in development. Modified from Mueller et al. (2016).

By the nature of our training, physiologists prefer to hold constant all but a single dependent environmental variable of interest (e.g., temperature, pH, oxygen), which is then controlled or allowed to vary. Yet, in the “real world,” of course, multiple variables are constantly changing concurrently. Thus, aquatic environments may be hypoxic because they are also warm, while high altitude terrestrial environments may carry have low oxygen and high radiation loads combined with low temperatures and humidities. In recognition of this, many developmental physiologists are beginning to expose developing animals to multiple rather than single changing stressors (Figure 3).

What is emerging from “multiple variable experiments” is that different organ systems and their system-specific physiologies have different critical windows for development. A challenge is to generate enhanced understanding of the interaction of development, time and environment through multi-variable experiments (Mueller, 2018).

### Riding the Wave of Technological Innovation

Developing animals are almost inevitably small, if not microscopic. Not surprisingly, then, miniaturization has long been a priority in instrumentation for developmental physiology (Burggren and Fritsche, 1995). Several recent technological developments have been aiding the assessment of physiological processes in progressively smaller (and thus earlier) developmental stages. Advances in imaging continues to improve our understanding of especial cardiac physiology of embryos, fetuses and larvae (Aguet et al., 2020; Salman and Yalcin, 2020; Lopez and Larina, 2021). The advent of microfluidics is contributing to our knowledge of embryonic physiology, especially as it pertains to *in vitro* embryo production (Wheeler and Rubessa, 2017; Sonnen and Merten, 2019). Organoids (“organs on a chip”) are increasingly being studied either on their own or as components of increasingly complex “organ” systems (Matsui et al., 2021). The study of organoids may yield extraordinary new insights into the assembly of the cells and tissues in early development. These and other emerging techniques typically produce vast amounts of data, often in the form of complex images. Not surprisingly, then, developmental biology, including developmental physiology, has begun to exploit to machine (“deep”) learning, which can analyze large data sets without direct human involvement and the associated risk of human-created error (Feltes et al., 2018; Villoutreix, 2021).

There are challenges associated with these and numerous other emerging technological innovations in the engineering processes, which continue to inexorably advance. Yet, the greater challenge may be providing broad access to these technologies and the training required to deploy them.

## Conclusions

In this short perspective on Grand Challenges in Developmental Physiology I have indicated directions in which the field of Developmental Physiology might (not necessarily should) move. Some of my predictions and suggestions will be on target, others less important than predicted, and new unimagined Challenges will emerged. What is crucial is that Developmental Physiology does not become complacent regarding its many achievements, continues to thrive on collaboration with other physiological disciplines, and ensures that its trainees are moving to the next stages of their careers with the skills and enthusiasm that continues to be a hallmark of developmental physiologists.

## Author Contributions

The author confirms being the sole contributor of this work and has approved it for publication.

## Conflict of Interest

The author declares that the research was conducted in the absence of any commercial or financial relationships that could be construed as a potential conflict of interest.
